# Guillain‐Barré syndrome associated with Coronavirus disease 2019: A case from Nepal

**DOI:** 10.1002/ccr3.5638

**Published:** 2022-03-23

**Authors:** Roshan Aryal, Shivaji Karki, Shreesti Rajbhandari, Bikram Prasad Gajurel, Ragesh Karn, Reema Rajbhandari, Sunanda Paudel, Niraj Gautam, Ashish Shrestha, Rajeev Ojha

**Affiliations:** ^1^ Maharajgunj Medical Campus Institute of Medicine Tribhuvan University Kathmandu Nepal; ^2^ Department of Neurology Institute of Medicine Tribhuvan University Kathmandu Nepal

**Keywords:** coronavirus, COVID‐19, Guillain‐Barré syndrome, neuropathy

## Abstract

Coronavirus disease 2019 (COVID‐19) has now spread widely after the outbreak since December 31, 2019. Guillain‐Barré syndrome is an immunological postinfectious neuropathy, which has been reported to be a rare but possible complication COVID‐19. We report a case of Guillain‐Barré syndrome associated with COVID‐19 in Nepal.

## Abstract 2

In this time of ongoing pandemic of COVID‐19, neurological manifestations might occur in the patients, one of which is Guillain‐Barré syndrome (GBS) that can cause significant morbidity to the patient and sometimes can even result in death.

## 1 INTRODUCTION

COVID‐19 is a beta coronavirus, which has now spread extensively all over the world after its novel outbreak from December 31, 2019.^1^ Common neurological manifestations of COVID‐19 include headache, dizziness, seizures, stroke, neuralgia, encephalitis, and hypogeusia.^2^, ^3^

Guillain‐Barré syndrome (GBS) is an immune‐mediated, postinfectious neuropathy characterized by progressive, symmetrical, and ascending weakness, with hyporeflexia or areflexia and with or without cranial nerve involvement.^4^, ^5^ Autonomic dysfunctions and sensory symptoms may or may not be present.^5^ Although the first reported case of GBS in Wuhan was proposed to be a parainfectious manifestation, it has also been reported as a rare but possible consequence of COVID‐19.^6^, ^7^, ^8^, ^9^ We report a case of GBS in a patient infected with COVID‐19 in Nepal.

## 2 CASE PRESENTATION

A 68‐year‐old man presented in the emergency, with acute symmetric progressive ascending quadriparesis, tingling sensation of the lower extremities, and dysphagia. Reverse transcription‐polymerase chain reaction (RT‐PCR) for severe acute respiratory syndrome coronavirus‐2 (SARS‐CoV‐2) was tested positive subsequently in the emergency department. Ten days prior to this, the patient had symptoms of myalgia, rhinorrhea, dysgeusia, and fever lasting for 5 days. After 5 days of his recovery, the patient suddenly developed difficulty in bearing weight in lower limbs while he woke up in the morning. Next day, he had difficulty holding food in his hands, though was able to feed by himself. Two days later he developed tingling, pin, and needle sensations over his lower extremities and lips. His weakness was also gradually worsening till 7 days after the onset of symptoms. During the presentation in emergency, the patient was bed‐bound and needed help for feeding and other functional activities. There was no swallowing difficulty and shortness of breath. Bowel and bladder were normal, and the patient is not diabetic.

On physical examination, the patient was conscious, afebrile with blood pressure measuring 160/100 mmHg, heart rate 100 beats/min, respiratory rate of 20 breaths/min, and oxygen saturation of 93 percent on room air. Facial nerve examination showed bilateral lower motor neuron type palsy (House‐Brackmann grade 3). Other cranial nerves were intact. No signs of meningeal irritation were present. Muscle strength examination showed a Medical Research Scale (MRC) of 2/5 in proximal, 3/5 in distal of upper extremities, 1/5 in proximal, and 2/5 in distal of lower extremities. Deep tendon reflexes were absent in all limbs. There was normal fine touch, pain, temperature, and vibration sensation in both upper and lower extremities.

The blood investigation results were as follows: total leukocyte count 8400/mm^3^ (neutrophils 72% and lymphocytes 24%); hemoglobin 14.2 g/dl; red blood cell 4.8 million/mm^3^; platelets 371,000/mm^3^; random sugar 6.7 mmol/L; sodium 135 mEq/L; and potassium 3.6 mEq/L. His renal and hepatic tests were within normal range. His prothrombin time was 13 s; prothrombin international normalized ratio 0.98; activated partial thromboplastin time 26.4 s; fibrinogen 432 mg/dl; fibrin degradation product (FDP) D‐dimer 0.36 mg/ml; and ferritin 382 μg/ml. Chest X‐ray and electrocardiograph findings were normal. Other laboratory tests including human immunodeficiency virus, syphilis, hepatitis B and C, and thyroid function test were unremarkable.

Eight days after the admission in COVID ward, the patient was shifted to the general ward. Cerebrospinal fluid analysis results showed a total leukocyte count of 5 cells (polymorph 0% and lymphocyte 100%), sugar 3.5 mmol/L, and protein 130 mg/dl. Due to unavailability of nerve conduction study in the inpatient settings, it could not be performed then.

With the strong clinical suspicion of Guillain‐Barré syndrome, the patient received 400 mg/kg/day intravenous immunoglobulin (IVIg) for 5 days while being admitted in the COVID ward. He also received gabapentin 300 mg two times a day for paresthesia in lower limbs. Patient's weakness of limbs gradually improved after IVIg treatment. During discharge, motor power of upper limbs improved to MRC of 4+ and 4 in lower limbs. In follow‐up in 1 month, weakness further improved and the patient is now able to walk without support and can do all his daily activities with only mild difficulties.

## 3 DISCUSSION

Respiratory symptoms like fever, difficulty in breathing, and cough are common manifestations due to the infection by beta coronaviruses, but neurological symptoms have also been reported.^10^, ^11^ Study by Mao et al.^12^ in Wuhan, China, has reported neurological symptoms in hospitalized patients with COVID‐19 with the conclusion of neurological manifestations being seen commonly in the patients with more Spartan COVID‐19 illness. Our patient satisfies the level 2 diagnostic certainty of Brighton criteria for GBS with all the features except the nerve conduction findings, which was not performed in our case.^13^ The Brighton diagnostic criteria for GBS can be found in Table 1.^13^

**TABLE 1:** Brighton diagnostic criteria for GBS^13^

Diagnostic criteria	Level of diagnostic certainty			
	Level 1	Level 2	Level 3	Level 4
The absence of alternative diagnosis for weakness	+	+	+	+
Diminished or absent deep tendon reflex in weak limbs	+	+	+	+/−
Monophasic course and time between onset and nadir, 12 h to 28 days	+	+	+	+/−
Bilateral and flaccid weakness of limbs	+	+	+	+/−
CSF cell count <50 cells/μl	+	+^a^	−	+/−
CSF protein concentration > normal value	+	+/−^a^	−	+/−
NCS findings consistent with one of the subtypes of GBS	+	+/−	−	+/−

Note

+: present; −: absent; +/−: present or absent.

Abbreviations: CSF, cerebrospinal fluid; GBS, Guillain‐Barré syndrome; NCS, nerve conduction study.

^a^ If CSF is not collected or results not available, nerve electrophysiology results must be consistent with the diagnosis Guillain‐Barré syndrome.

Prior to GBS findings, our case had a characteristic course of viral symptoms for 5 days. Meanwhile, the duration of onset of viral illness and neurological manifestations has ranged from 5 to 24 days.^14^ Five cases of COVID‐19 developing GBS 5–10 days after infection were reported by Toscano et al.^7^ However, simultaneous occurrences of respiratory and neurological symptoms have also been reported.^15^, ^16^ In Iran, three patients were diagnosed with GBS during the active phase of COVID‐19.^17^

Our patient did not have respiratory failure and also did not require mechanical ventilation during the course of his hospital stay. A meta‐analysis summarizing 42 cases of GBS in the setting of COVID‐19 from January to August of 2020 reported 14 of these patients had respiratory failure, and 12 of them required mechanical ventilation.^18^ With some overlap in the included studies, another meta‐analysis presented 61 cases of GBS associated with COVID‐19, of which 23 needed intensive care unit (ICU) admission, with 17 requiring mechanical ventilation.^19^ Furthermore, a systematic review comprising 12 patients described five patients having symptom remission or mild persistent symptoms, four of them requiring critical care and one death.^20^

Guillain‐Barré syndrome is an immune‐mediated disorder triggered by an infection or immune stimulus with the initiation of an uncharacteristic immune reaction to peripheral nerves as a result of molecular mimicry.^21^ COVID‐19 creates an immune‐mediated course due to stimulation of various inflammatory cells and thereby leading to production of inflammatory cytokines.^22^ It is still uncertain whether GBS due to COVID‐19 is due to antibody production against specific gangliosides as seen in some forms of GBS or T‐cell‐mediated actions or straight neuroinvasive events.^23^, ^24^ There is a need for further investigations about the development of GBS in patients with COVID‐19.

## CONFLICT OF INTEREST

The authors declare that there are no conflicts of interest.

## AUTHOR CONTRIBUTIONS

(RO) Rajeev Ojha, (BPG) Bikram Prasad Gajurel, (RK) Ragesh Karn, (RR) Reema Rajbhandari, (SP) Sunanda Paudel, (NG) Niraj Gautam, and (AS) Ashish Shrestha involved in the patient care team and contributed to the collection of case information. (RO) Rajeev Ojha contributed to conceptualization and editing of the manuscript. (RA) Roshan Aryal, (SK) Shivaji Karki, and (SR) Shreesti Rajbhandari collected all the required case information and reports and reviewed the literature and contributed in writing and editing the manuscript. All authors read and approved the final manuscript.

## ETHICAL APPROVAL

This study did not include experiments on animals or humans. The patient gave consent to use his details for this case study.

## CONSENT

Written informed consent was obtained.

## Data Availability

The data that support the findings of this study are available from the corresponding author, upon reasonable request.
